# Nocebo-Prone Behaviour in Patients with Autoimmune Rheumatic Diseases during the COVID-19 Pandemic

**DOI:** 10.31138/mjr.31.3.288

**Published:** 2020-09-21

**Authors:** George E. Fragoulis, Gerasimos Evangelatos, Aikaterini Arida, Vasiliki-Kalliopi Bournia, Kalliopi Fragiadaki, Anastasios Karamanakos, Evrydiki Kravvariti, Katerina Laskari, Stylianos Panopoulos, Maria Pappa, Dimos D. Mitsikostas, Maria G. Tektonidou, Petros P. Sfikakis

**Affiliations:** 1 First Department of Propaedeutic Internal Medicine, Joint Rheumatology Program, National and Kapodistrian University of Athens Medical School, “Laiko” General Hospital, Athens, Greece; 2 First Neurology Department, Aeginition Hospital, Medical School, National and Kapodistrian University of Athens, Athens, Greece

**Keywords:** COVID-19, nocebo, autoimmune rheumatic diseases

## Abstract

**Background:**

The COVID-19 pandemic is associated with emotional distress and significant disruptions in health-care services. These are key players in the development of nocebo phenomena. We aimed to investigate nocebo-prone behaviour in patients with autoimmune rheumatic diseases (ARD) amid the COVID-19 pandemic-associated lockdown.

**Methods:**

Consecutive patients were telephone-interviewed during the COVID-19 pandemic in Greece. Clinical and socioeconomic characteristics (eg, level of education) were recorded. For nocebo behaviour, a four-item validated questionnaire (Q-No, cut-off score>15), was used. Results were compared with pre-COVID-19 Q-No scores collected from patients followed-up in our department.

**Results:**

Nocebo behaviour was detected in 51/500 (10.2%) individuals. In patients with nocebo behaviour, use of anti-hypertensives was less common (17.6% vs 31.8%, p=0.04), but a higher level of education was more common (58.8% vs 35.9%, p=0.002), compared with patients with Q-No score ≤15; the latter retained statistical significance in multivariate regression analysis (p=0.009, OR [95%CI]: 2.29, [1.23–4.25]). Total Q-No scores were higher in the COVID-19-period compared to the pre-COVID-19 era [median (range); 12 (4–20) vs 11 (4–20), p=0.02]. Among 78 patients with available Q-No questionnaires in the pre-COVID-19 era, 11 (14.1%) displayed nocebo behaviour, which increased to 16 (20.5%) amid the COVID-19 pandemic. Interim development of nocebo behaviour was also associated with higher educational level (p=0.049, OR: 3.65, 95%CI: 1.005–13.268).

**Conclusion:**

A considerable proportion of ARD patients manifested nocebo-prone behaviour during the COVID-19 pandemic, which was more common among those with high educational level.

## INTRODUCTION

The coronavirus disease 2019 (COVID-19) pandemic has significantly affected our everyday life in many aspects. Several public health restrictive measures, such as social distancing and home isolation, were taken to impede virus spreading. Apparently, outpatient clinics, including appointments with rheumatologists, were heavily affected and therefore patient-doctor interaction has been severely disrupted. Besides, the COVID-19 pandemic is being linked with emotional distress similar to that seen in other crises.^1^ One could speculate that the psychological effects of COVID-19 pandemic could be even more pronounced in patients with autoimmune rheumatic diseases (ARD), since many of the latter are on treatment with immunosuppressives.

The aforementioned factors have been characterised as key players in the nocebo effect.^2^ The latter has been described as the opposite of the placebo effect, and denotes the non-intentional negative physical symptoms in response to a medical treatment, which are not a result of pharmacologic action, but occur solely due to negative expectations on behalf of the patient. Neuro-behavioural mechanisms seem to associate with its occurrence, but are so far poorly understood.^3^ In recent years, nocebo effects have been recognized as a cause of treatment non-adherence and worse disease outcomes,^2^ which is especially relevant in patients with ARD switching to treatment with biosimilars;^4–6^ several studies in this field have incorporated interviews and questionnaires aiming to quantify and/or reduce nocebo-prone behaviour.^7,8^

Herein, we aimed to investigate nocebo-prone behaviour in ARD patients during the COVID-19 pandemic and whether this has changed compared to the pre-COVID-19 era.

## MATERIALS AND METHODS

### Study population and recorded parameters

Five hundred patients with various ARDs were interviewed via telephone from rheumatologists of our department during the COVID-19 pandemic period in Greece. In particular, interviews were conducted from 14/04/2020 to 22/04/2020. Patients included in this study had been followed-up for at least 6 months in the outpatient rheumatology clinics of our academic center. They were consecutively enrolled, following an alphabetical order of their surname as recorded in our files. Patients with osteoarthritis, crystal arthropathies, undifferentiated rheumatic disease, metabolic bone disease, and fibromyalgia as primary diagnosis, were excluded. The following demographic and clinical characteristics were recorded: age, sex, cohabitation (defined as living or not with a partner), region of residence (urban, semi-urban, rural), level of education (primary, secondary, higher), employment status, and disease duration. Current treatment for ARDs and for the presense of co-morbidities (hypertension, hyperlipidemia, coronary heart disease, diabetes mellitus, depression, anxiety) were also recorded.

For detection of nocebo behaviour, a validated questionnaire (Q-No),^7^ composed of four questions was performed (Supplementary Table 1). These questions referred to the period of the COVID-19 pandemic in Greece, starting on 26 February 2020 (first reported case). Nocebo behaviour was cosidered to be present when score of this questionaire was above 15.^7^ In the pre-COVID-19 era, data for nocebo behaviour were collected prospectively from patients who were routinely followed-up in our outpatients’ rheumatology clinics.

**Table 1.** Total nocebo score as well as values for each question of the Q-No questionnaire. Q-No: Questionnaire Nocebo, Q: questionnaire

	**Nocebo Behaviour (n=51)**	**Non-nocebo behaviour (n=449)**	**p-value**
Q1, median (range)	5 (2–5)	3 (1–5)	<0.0001
Q2, median (range)	3 (1–5)	1 (1–5)	<0.0001
Q3, median (range)	5(3–5)	3 (1–5)	<0.0001
Q4, median (range)	5 (3–5)	2 (1–5)	<0.0001
Sum, median (range)	17 (16–20)	10 (4–17)	<0.0001

Also, as part of another recently published study by our group,^9^ specific questions, with predefined answer-options were asked: discontinuation or tapering of the medication being received for ARDs, possible reasons that led to drug discontinuation (including but not confined to: fear of immunosuppresion and lack of reourses/drug shortage), whether advice was received from a clinician or other sources, possible symptomatology compatible with COVID-19 infection, testing for COVID-19, and subjective assessment of the activity of their disease (Supplementary Table 2).

**Table 2.** Comparison between patients with nocebo-behaviour during COVID-19 pandemic and those without. SD: standard deviation, n: number, CHD: coronary heart disease, DM: diabetes mellitus, COPD: chronic obstructive pulmonary disease, HCQ: hydroxychloroquine.

	**Nocebo Behaviour (n=51)**	**Non-nocebo behaviour(n=449)**	**p-value**
	Demographics and clinical		
Age (years), mean ± SD	50.7 ± 14.7	54.1 ± 15.4	0.137
Sex (female), n (%)	36 (70.6)	330 (73.5)	0.622
Disease duration (years), mean ± SD	9.7 ± 8.0	10.2 ± 9.5	0.967
Co-habitating, n (%)	28 (54.9)	301 (67.0)	0.09
Region residence (urban), n (%)	44 (86.3)	374 (83.3)	0.693
Employment, n (%)	49 (96.1)	422 (94.0)	0.756
Higher education level, n (%)	30 (58.8)	161 (35.9)	**0.002**
Hypertension, n (%)	9 (17.6)	143 (31.8)	**0.04**
CHD, n (%)	2 (3.9)	35 (7.8)	0.410
Dyslipidaemia, n (%)	3 (5.8)	69 (15.4)	0.09
Osteoporosis, n (%)	7 (13.7)	58 (12.9)	0.827
Depression, n (%)	8 (15.7)	76 (16.9)	1.000
Anxiety, n (%)	2 (3.9)	13 (2.9)	0.658
DM, n (%)	2 (3.9)	41 (9.1)	0.293
COPD, n (%)	2 (3.9)	9 (2.0)	0.311
On biologics, n (%)	23 (45.1)	224 (49.9)	0.556
On HCQ, n (%)	10 (19.6)	124 (27.6)	0.725
	COVID-19-pandemic related features		
Drug discontinuation due to fear, n (%)	1 (2.0)	10 (2.2)	1.000
Drug discontinuation duo to lack of resources, n (%)	1 (2.0)	18 (4.0)	0.709
Covid-19 symptoms, n (%)	4 (7.8)	35 (7.8)	1.000
Consultation of a clinician, n (%)	16 (31.4)	108 (24.1)	0.304
Outcome (worse), n (%)	10 (19.6)	67 (14.9)	0.412

This study was conducted according to the Declaration of Helsinki and was approved by the Scientific Council of our hospital.

### Statistical analysis

Patients who manifested or developed nocebo behaviour were compared to those who did not. Two-sided Fisher’s exact and Mann-Whitney tests were used to compare categorical and continuous characteristics, respectively. Wilcoxon matched paired test was used for paired samples. Binary logistic regression analysis was performed using “Positive Nocebo behaviour”as dependent variable, and sex, age, living alone, urban area of residence, unemployment, level of education, disease duration, presence of arterial hypertension, coronary heart disease, hyperlipidemia, depression, anxiety, diabetes mellitus and chronic obstructive pulmonary disease (COPD) as independent variables. Statistical analysis was performed using SPSS 21.0 (Armonk, NY: IBM Corp, USA).

## RESULTS

### Nocebo behaviour during the COVID-19 pandemic

Five hundred patients with various ARDs were included. As previously described,^9^ these were: inflammatory arthritis: 52.4%, systemic lupus erythematosus: 16%, systemic sclerosis: 11%, antiphospholipid syndrome: 3.6%, Sjögren syndrome: 1.2%, polymyalgia rheumatica: 1.2%, vasculitis: 9.4%, auto-inflammatory diseases: 5.2%.

During the COVID-19 pandemic, nocebo behaviour was detected in 51/500 (10.2%) patients. Their median (range) nocebo score, 17 (16–20) was significantly higher compared to those who did not display nocebo behaviour (10 [4–17], p<0.0001). In addition, statistically significantly higher values for all individual questions of the score (all for p<0.0001) were observed in patients with nocebo behaviour (Table 1).

### Associations of Nocebo behaviour and demographic, clinical and COVID-19-pandemic-related characteristics

Comparing patients who had nocebo behaviour with those who did not, we showed that the former were less frequently on anti-hypertensives (17.6% Vs 31.8%, p=0.04) and had more frequently high level of education (58.8% Vs 35.9%, p=0.002). No other differences were detected regarding demographic and clinical characteristics. COVID-19-pandemic related features, including, but not confined to, discontinuation due to fear and/or due to lack of resources, were comparable between the two groups (Table 2). Multivariate regression analysis confirmed that higher level of education was associated with nocebo behaviour (p=0.009, OR [95%CI]: 2.29, [1.23–4.25]).

### Change in Nocebo behaviour during Covid-19-pandemic

Regarding the pre-COVID-19 era, data about nocebo effect were available for 78 of these 500 patients, 11 of which (14.1%) had displayed nocebo behaviour. This proportion did not differ significantly from the respective during the pandemic (p=0.324).

However, when nocebo scores were analysed as continuous variables, total Q-No scores in the 78 patients were higher in the COVID-19-period compared to the pre-COVID-19 era (median [range]; 12 [4–20] Vs 11 [4–20], p=0.02). The same was the case for question 3 (“I ask my physician for potential adverse effects of the medication he/she gives me”) of the Q-No questionnaire (median [range]; 3 [1–5] Vs 2 [1–5], p=0.02) (Table 3).

**Table 3.** Total nocebo score and values for each question of the Q-No questionnaire. Comparison between pre-Covid-19 era and Covid-19-pandemic period. Q-No: Questionnaire Nocebo, Q: question

	**pre-Covid-19 era**	**Covid-19-pandemic period**	**p-value**
Q1, median (range)	4 (1–5)	4 (1–5)	0.212
Q2, median (range)	2 (1–5)	2 (1–5)	0.189
Q3, median (range)	2 (1–5)	3 (1–5)	**0.02**
Q4, median (range)	3 (1–5)	3 (1–5)	0.07
Sum, median (range)	11 (4–20)	12 (4–20)	**0.02**

Amid the COVID-19-pandemic period, 16/78 (20.5%) of patients developed nocebo behaviour; in 7/78 (8.9%), Q-No scores were initially over 15 and dropped below the cut-off for nocebo behaviour detection, while in the rest of them, no change was noticed.

In the univariate analyses, new development of nocebo behaviour was associated with higher baseline nocebo scores (p=0.05), younger age (p=0.03), and higher education level (p=0.03). Only the latter retained its statistical significance (p=0.049, OR: 3.65, 95% confidence interval; 1.005 – 13.268) after correcting for age, sex, baseline nocebo scores and treatment with biologics or hydroxychloroquine. COVID-19-pandemic related features were comparable between patients who developed nocebo behaviour and those who did not (Table 4).

**Table 4.** Comparison between patients who newly developed nocebo behaviour during Covid-19-pandemic, and those who did not. SD: standard deviation, n: number, IHD: ischemic heart disease, DM: diabetes mellitus, COPD: chronic obstructive pulmonary disease, HCQ: hydroxychloroquine.

	**Development of new Nocebo behaviour(n=16)**	**Stable/attenuated Nocebo behaviour (n=62)**	**p-value**
	Demographics and co-morbidities		
Age (years), mean ± SD	49.7 ± 14.8	57.6 ± 15.6	**0.03**
Sex (female), n (%)	11 (68.7)	40 (64.5)	1.000
Disease duration (years), mean ± SD	12.5 ± 7.9	10.1 ± 9.4	0.117
Co-habitating, n (%)	9 (56.3)	42 (67.7)	0.395
Region residence (urban), n (%)	14 (87.5)	43 (69.3)	0.210
Employment, n (%)	16 (100.0)	56 (90.3)	0.336
Higher education level, n (%)	9 (56.3)	16 (25.8)	**0.033**
Noceboprevious score	12.4 ± 3.2	10.6 ± 4.0	**0.05**
Hypertension, n (%)	2 (12.5)	24 (38.7)	0.07
IHD, n (%)	1 (6.25)	5 (8.1)	1.000
Dyslipidaemia, n (%)	0 (0.0)	10 (16.1)	0.112
Osteoporosis, n (%)	2 (12.5)	11 (17.7)	1.000
Depression, n (%)	3 (18.8)	12 (19.3)	1.000
Anxiety, n (%)	1 (6.25)	0 (0.0)	0.205
DM, n (%)	2 (12.5)	7 (11.3)	1.000
COPD, n (%)	0 (0.0)	1 (1.6)	1.000
On Biologics, n (%)	10 (62.5)	37 (59.6)	1.000
On HCQ, n (%)	2 (12.5)	9 (14.5)	1.000
	COVID-19-pandemic related features		
Drug discontinuation due to fear, n (%)	0 (0.0)	2 (3.2)	1.000
Drug discontinuation due to lack of resources, n (%)	0 (0.0)	7 (11.2)	0.334
Covid-19 symptoms, n (%)	0 (0.0)	4 (6.4)	0.576
Consultation of a clinician, n (%)	5 (31.3)	14 (22.3)	0.520
Outcome (worse), n (%)	4 (25.0)	8 (12.9)	0.254

## DISCUSSION

The COVID-19 pandemic, along with the measures that came with that, affected significantly our every-day life and activities. As observed in other disasters, mental health can be also affected.^1,10^ Although hard evidence is lacking, it is not irrational to say that this is expected to be more pronounced in patients with chronic diseases, such as ARDs.^1,2^ Indeed, qualitative data from a recent study showed that patients with ARDs identified emotional disturbance during the pandemic as a key issue.^11^ We hypothesised that alterations in the mental health status along with disturbances in the health-care setting would lead to nocebo-phenomena.

We showed that nocebo behaviour was observed in 10% of our cohort which was representative of the patients who are followed-up in our university rheumatology clinic. Interestingly, patients with a high educational level were more prone to nocebo behaviour, while patients with concomitant hypertension exhibited nocebo characteristics less frequently that those without hypertension. Regarding the former, previous data is controversial. Although it has been shown that educational status might not have any impact on nocebo effect in patients with cancer,^12^ Hoffman et al. have shown that cancer patients that have some college education experience more drug side effects than high school graduates or people that have not finished high school.^13^ Moreover, nocebo effect is more likely to be triggered during oral drug provocation tests in subjects with high education.^14^ It is possible that individuals with higher education levels might be more suspicious to drug-related adverse events. Interestingly, a recent study from China showed that people with higher education level also tended to have more psychological distress during the COVID-19 pandemic.^15^ On the other hand, there is a lack of data regarding the nocebo effect in patients under anti-hypertensives. As these drugs are effective and generally well-tolerated, one could hypothesise that they can strengthen patient’s optimism about drugs. This could indirectly decrease the nocebo effect, as optimists are less likely to follow the nocebo expectation.^16,17^

Comparing the nocebo susceptibility classification before and during the COVID-19 outbreak, we found that the proportion of patients with “positive nocebo behaviour” did not differ significantly. However, an increase in the absolute number of nocebo score was noticed. This increase was mainly attributed to the trend of increasingly asking the treating physician about the adverse events of the drugs receiving, as depicted in the question 3 of Q-No. It can be hypothesised that on the basis of a pandemic, due to the fear of immunosuppression, ARD patients refer more to their rheumatologist regarding possible drug adverse events.

We also showed that 20% of the patients developed “positive nocebo behaviour” group amid COVID-19 pandemic. This was associated with higher baseline nocebo score, younger age, and higher educational levels, with the latter retaining its statistical significance after adjustments.

We acknowledge that this study has limitations. Firstly, the nocebo behaviour was assessed differently before and during the pandemic. The Q-No questionnaire was filled by the patient itself in the outpatient clinic visit before the pandemic, but during the outbreak the questionnaire was filled by a rheumatologist after a phone call with the patient. Nevertheless, this was inevitable due to the quarantine measures. Secondly, the Q-No questionnaire has not been validated in patients with ARDs, as the clinical significance of nocebo effects in this population has been appraised only recently. To our knowledge, this is the first study to assess nocebo behaviour in ARD patients, and also in individuals in general amid the COVID-19 pandemic.

In conclusion, nocebo behaviour has been developed in a considerable proportion if ARD patients during COVID-19 pandemic. This seems to owe to patients’ concerns about drug reactions. Nocebo behaviour occurred or developed more frequently in highly-educated individuals. Rheumatologists should be aware of the negative psychological impact of the COVID-19 pandemic in ARD patients of specific characteristics. It remains to be seen whether this will be mirrored in clinical outcomes over the next months.

## Figures and Tables

**Supplementary Table 1.** Questions asked to detect nocebo behaviour. *score of 1-5 for each of the four (A–D) questions. Total score (A+B+C+D) ranges from 4–20 (7).

**Questions**	**Possible answers**
A. I read the summary of product characteristics (SPC) before I take any medication	*1. Never, 2. Rarely, 3. Sometimes, 4. Often, 5. Always
B. I have stopped in the past medication, due to side effects	
C. I ask my doctor for possible side effects of the drug I am on	
D. Is of great importance to me, the side effects reported in the SPC	

**Supplementary Table 2.** Questions asked during the telephone survey. ^start of pandemic for Greece, is considered the 26^th^ of February, when the first case of covid-19 was confirmed. *: patients who did not /get prescription, who could not go to the pharmacy, # discontinuation, reduction, escalating

**Questions**	**Possible answers**
**A1.** Did you discontinue/taper any of your medications received for rheumatic diseases during Covid-19 pandemic^?	YES / NO
If YES, did you have any reason?	1. “scared of infections”, 2. “forgot to take my medication”, 3. “lack of resources”*, 4. “respiratory infection”, 5. “adverse events or non-efficacy”, 6. “other” (free text)
**A2.** Did you consult anyone for changing^#^, your medication	YES / NO
If YES, whom?	1. Family doctor, 2. Rheumatologist, 3. Public health authorities, 4. Media, 5. Other patients/forums, 6. Relatives/friends, 7. Other (free text)
**B1.** Did you have any of the following symptoms: cough, fever, dyspnoea, olfactory loss, diarrhoea, myalgias, severe fatigue	YES / NO
**B2.** Test for Covid-19	Positive/Negative/Not done
**C.** How do you feel about your rheumatic disease, over the last month?	1. Significantly improved, 2. Mildly improved, 3. Stable, 4. Mildly deteriorated, 5. Significantly deteriorated.

## ABBREVIATIONS

ARD: Autoimmune rheumatic diseases
COPD: Chronic obstructive pulmonary disease
COVID-19: Coronavirus disease 2019
OR: Odds ratio
Q-No: Nocebo questionnaire
